# Supplementary material for: SLC45A4 encodes a peroxisomal putrescine transporter that promotes GABA de novo synthesis
Source: Nat Commun. 2025 Nov 20;16:10198. doi: 10.1038/s41467-025-62721-x (PMC12634670; doi:10.1038/s41467-025-62721-x)
Supplement: Supplementary file 1 — Supplementary Information [file 41467_2025_62721_MOESM1_ESM.pdf]

Supplementary Table 1: Correlation between 464 SLCs gene expression (RPKM) and 225 metabolites from 898 human cancer line compiled in CCLE database.

Supplementary Figure 1. Heatmap of SLCs gene expression (RPKM) correlated with metabolite measurements.

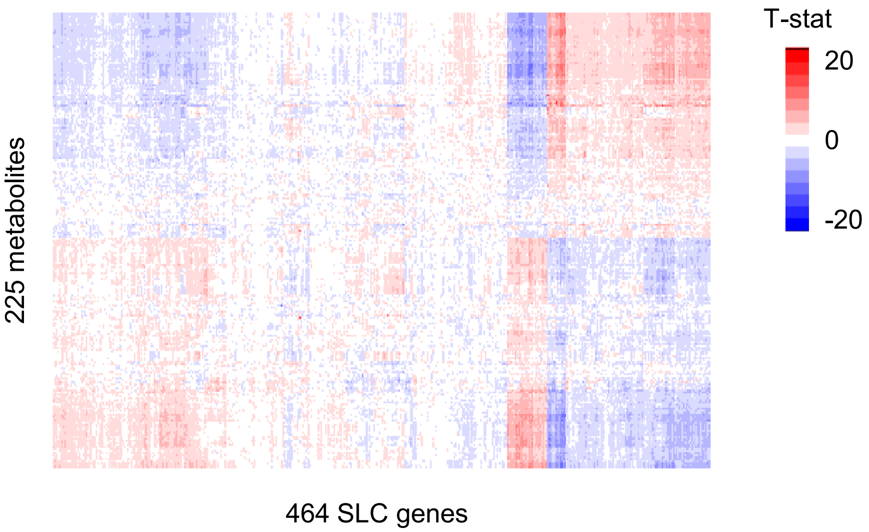

Supplementary Figure 2. Correlation between SLC6A8 expression levels and cellular Creatine levels

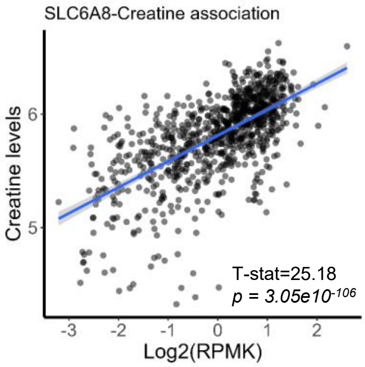

Supplementary Figure 3. Correlations between cellular GABA levels and **a.** known GABA transporters, **b**. synthesis enzymes and **c**. ODC1.

a. GABA transporters

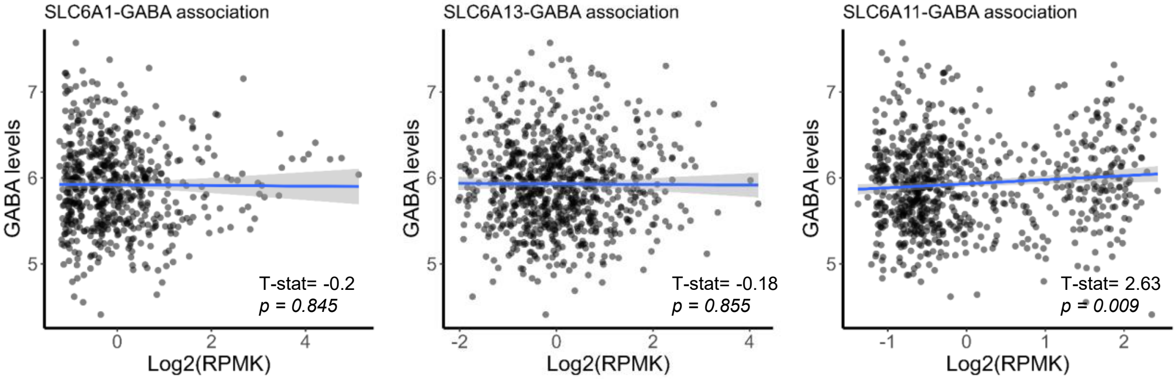

b. GABA canonical synthesis enzymes

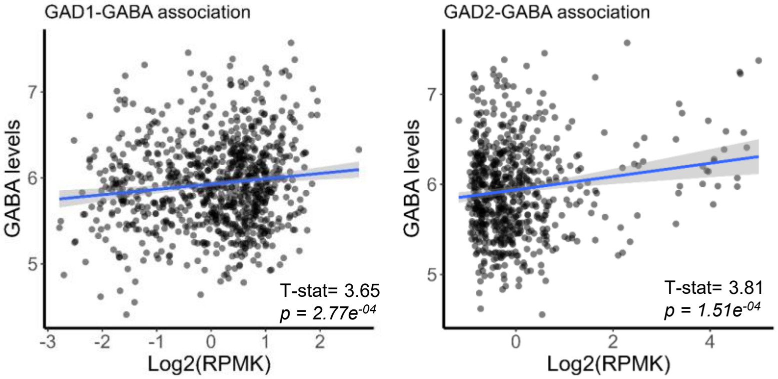

c. Ornithine Decarboxylase

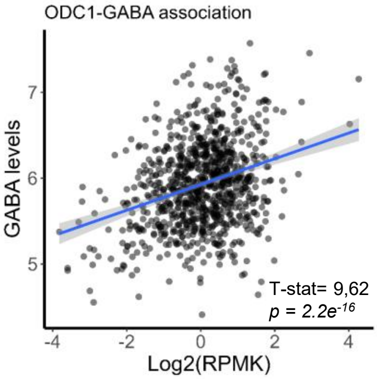

Supplementary Figure 4. GABA quantitation in lysates and media from A549, H1299 and HepG2 cells.

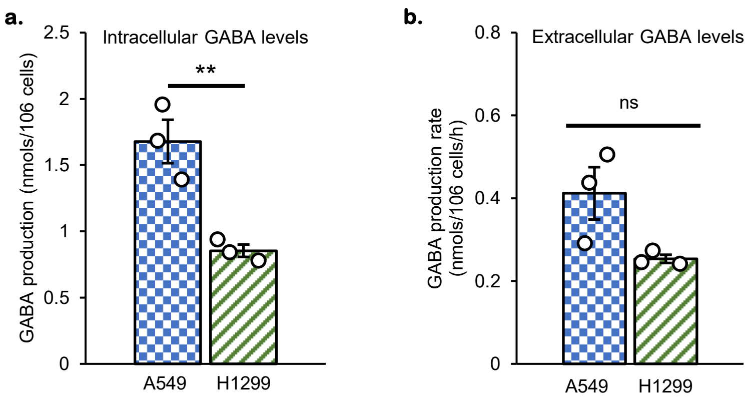

Supplementary Figure 4. GABA quantitation in lysates and media from A549, H1299 and HepG2 cells.
Endogenous GABA concentration (^{12}C-GABA) in lysates (**a.**) and media (**b.**) from A549 et H1299 cells measured by LC-MS and $^{13}C_4$-GABA as internal standard.

Supplementary Figure 5. SLC45A4 is not a sucrose transporter and GABA is not derived from glucose.

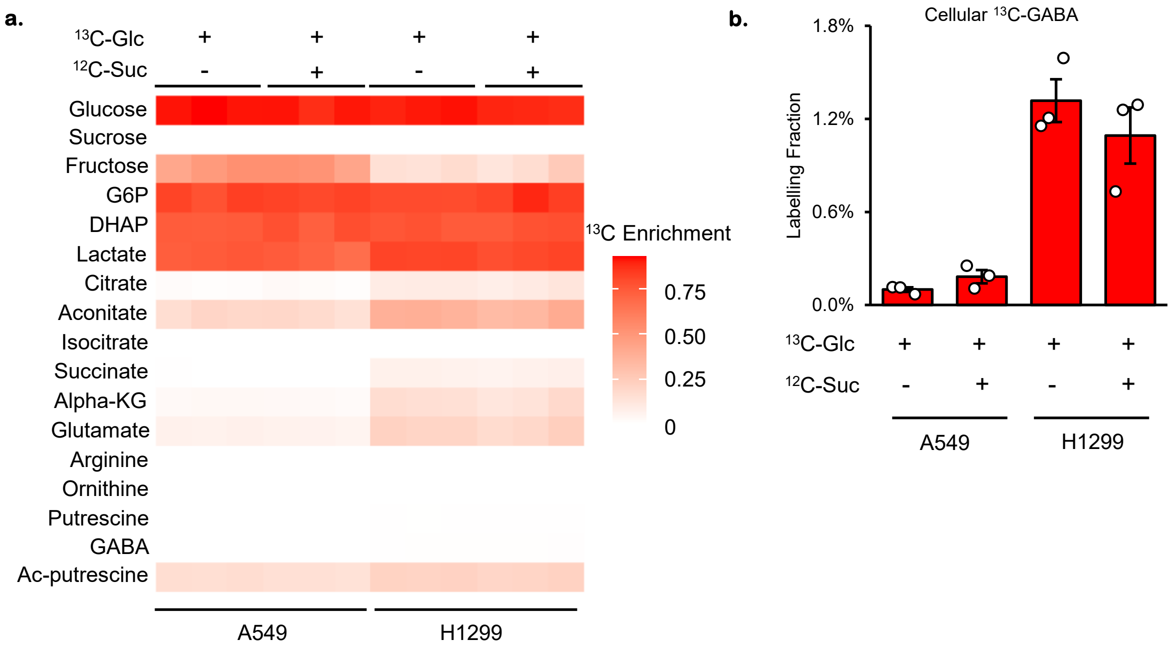

Supplementary Figure 5. SLC45A4 is not a sucrose transporter and GABA is not derived from glucose.
a. Enrichment fractions of A549 and H1299 cells supplemented with $^{13}C_6$-glucose in absence (-) or presence (+) of unlabeled sucrose (^{12}C-sucrose). **b**. Cellular GABA fractions in A549 and H1299 cells supplemented with $^{13}C_6$-glucose in absence (-) or presence (+) of unlabeled sucrose (^{12}C-sucrose).

Supplementary Figure 6. Mass spectrum of GABA in H1299 cells

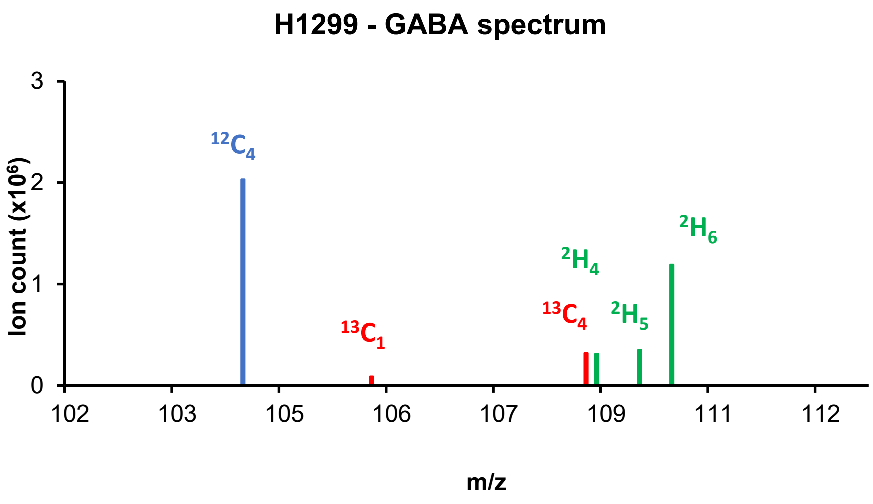

Supplementary Figure 6. Mass spectrum of GABA in H1299 cells simultaneous labeled from 1.15 mM $^{13}C_6$-arginine and 0.2 mM $^{2}H_6$ ornithine and cultured in heat-inactivated dialyzed FBS.

Supplementary Figure 7. ODC1 overexpression (OE-hODC1) increases a. intracellular and b. extracellular putrescine

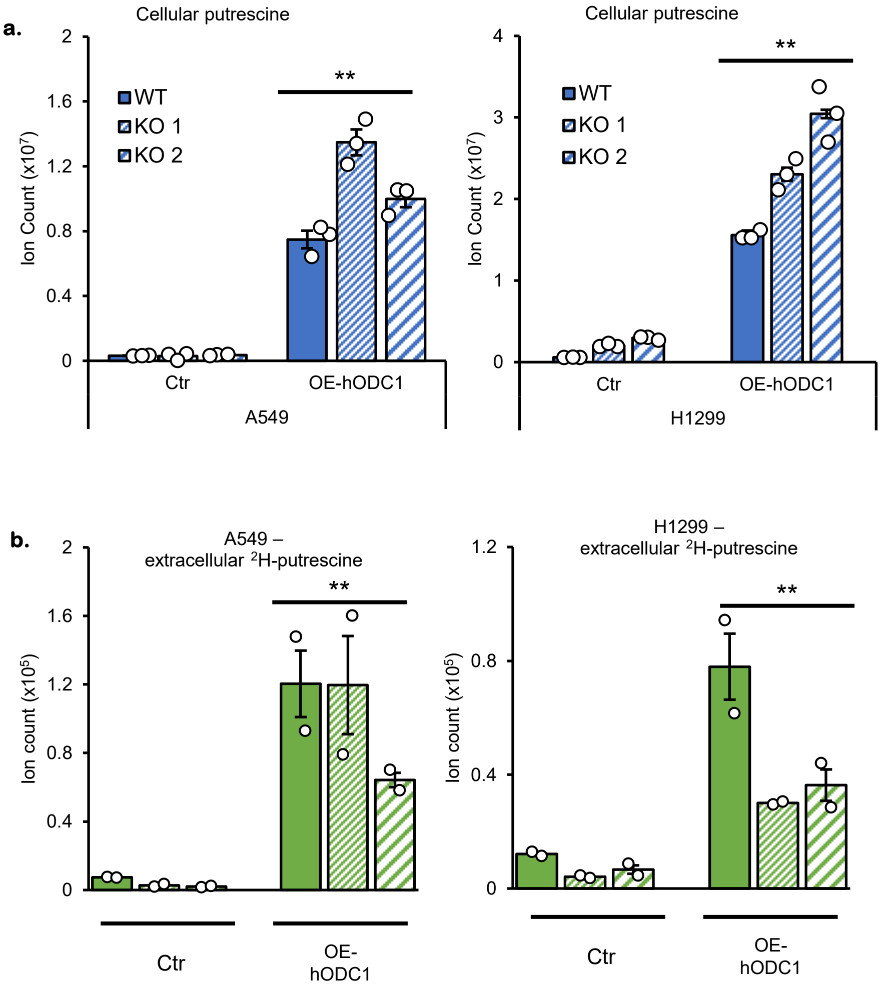

Supplementary Figure 7. ODC1 overexpression increases **a**. intracellular and **b.** extracellular putrescine. Cellular (top panel) and media (bottom panel) levels of putrescine in A549 (left) and H1299 (right) WT and their SLC45A4-KO cells (KO 1 & KO 2) overexpressing empty vector (Ctr) or human-ODC1 (OE-hODC1).

Supplementary Figure 8. Intracellular GABA synthesis depends on intracellular DAOs.

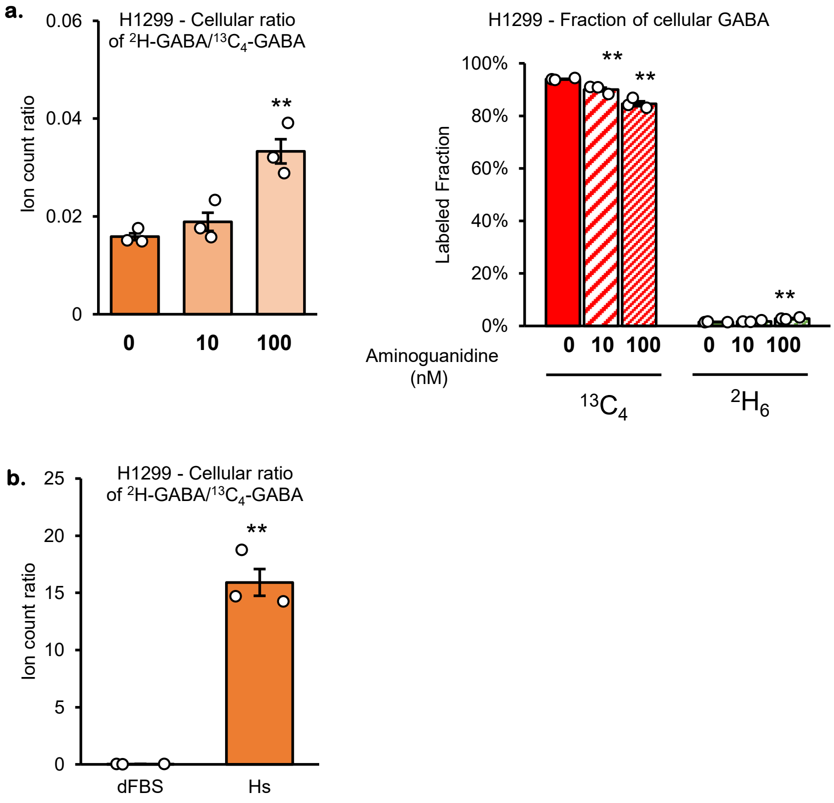

Supplementary Figure 8. Intracellular GABA synthesis depends on intracellular DAOs. **a.** ^{2}H/^{13}C-GABA ratio (left panel) and labeled fraction (right panel) in H1299 WT cells in absence (0) or presence of 10 nM or 100 nM of DAOs irreversible inhibitor aminoguanidine. **b.** ^{2}H/^{13}C-GABA ratio in H1299 WT cells cultured in regular dialyzed heat inactivated serum (dFBS) or Horse serum (Hs)

Supplementary Figure 9. Anti-SLC45A4 from Invitrogen specifically detects overexpressed SLC45A4-HA.

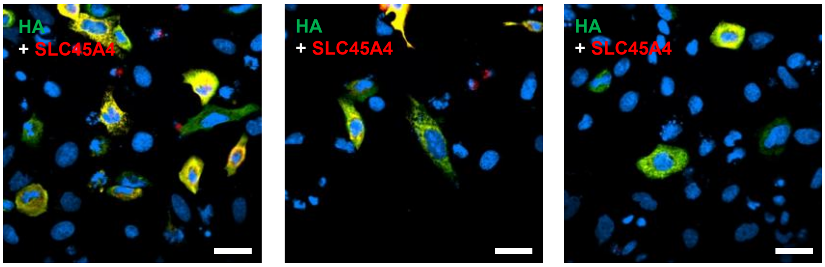

Supplementary Figure 9. Anti-SLC45A4 from Invitrogen specifically detects overexpressed SLC45A4-HA.
Immunofluorescent detection of SLC45A4-HA. SLC45A4, red; HA-tag, green. Micrographs are representatives from 3 independent experiments. Scale bar 10 μm.

Supplementary Figure 10. SLC45A4 is not a plasma membrane transporter neither localized in the Golgi apparatus, the endoplasmic reticulum or the mitochondria

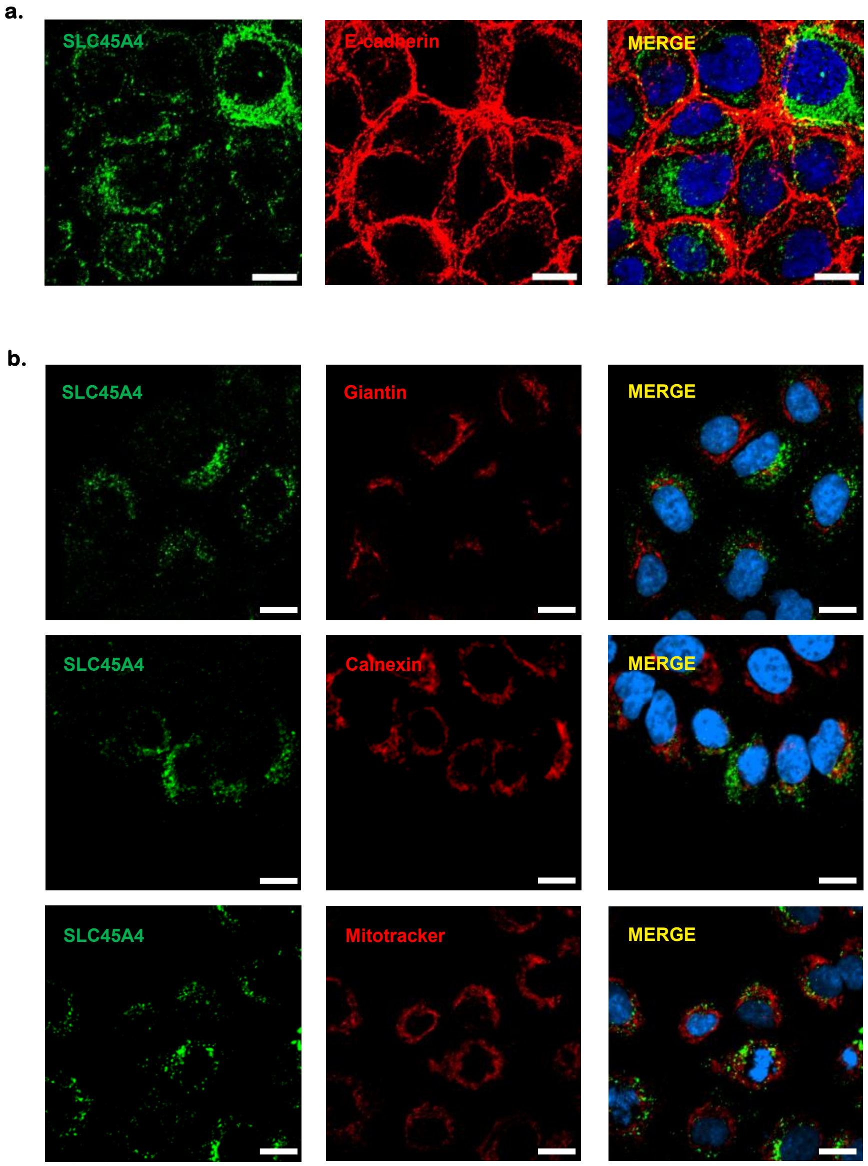

Supplementary Figure 10. SLC45A4 is not a plasma membrane transporter neither localized in the Golgi apparatus, the endoplasmic reticulum or the mitochondria Immunofluorescent detection of SLC45A4 subcellular localization. **a.b.** SLC45A4 endogenous, green; **a.** plasma membrane marker, e-cadherin, red; **b**. organellar markers, Giantin (Golgi apparatus), Calnexin (Endoplasmic reticulum), and Mitotracker (Mitochondria) red. Micrographs are representatives from 3 independent experiments. Scale bar 10 µm.

Supplementary Figure 11. SLC45A4 protein is enriched with peroxisomal proteins

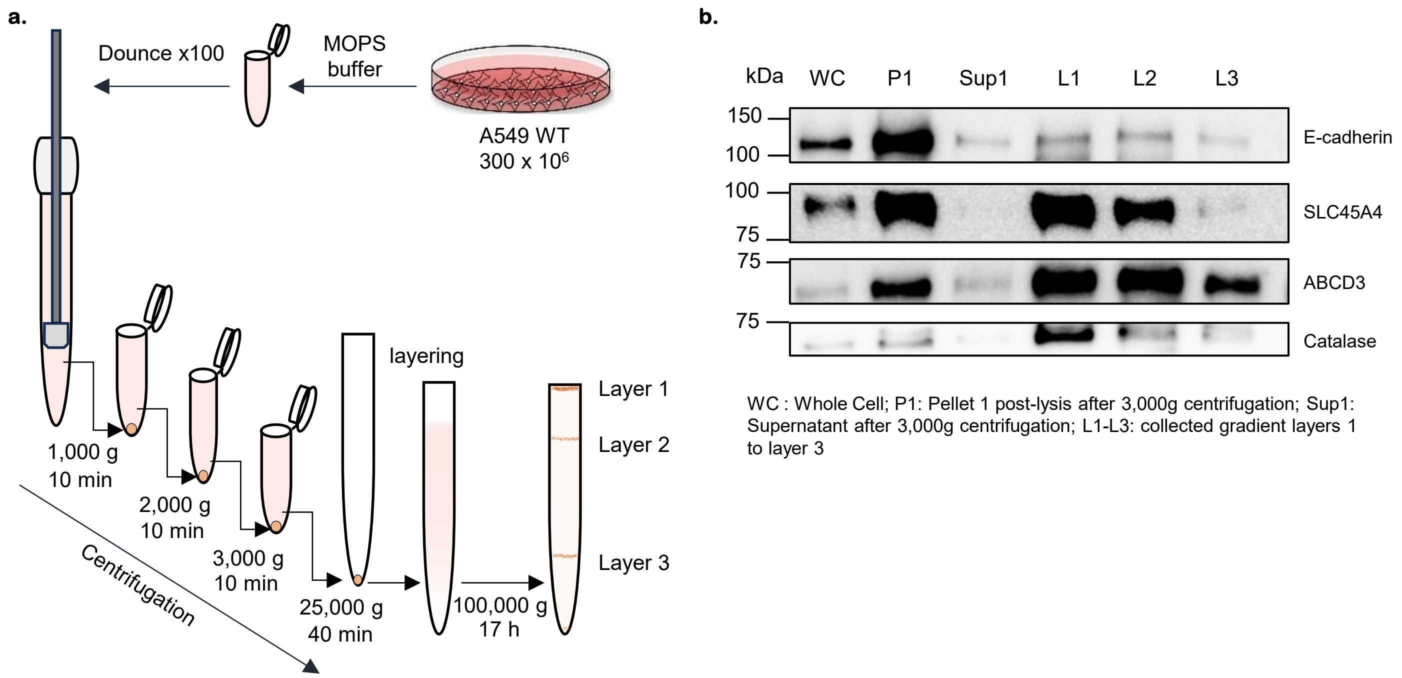

Supplementary Figure 11. SLC45A4 protein is enriched with peroxisomal proteins. **a.** A549 WT cells were used to isolate peroxisomes following the protocol adjusted from PEROX1 (Sigma). Image adapted from Servier Medical Art, licensed under CC BY 4.0. **b.** SDS-PAGE of A549 cells sub-fractionated using differential centrifugation and density gradient.
