# Supplementary material for: SLC45A4 encodes a peroxisomal putrescine transporter that promotes GABA de novo synthesis
Source: Nat Commun. 2025 Nov 20;16:10198. doi: 10.1038/s41467-025-62721-x (PMC12634670; doi:10.1038/s41467-025-62721-x)
Supplement: Supplementary file 2 — Description of Additional Supplementary Files [file 41467_2025_62721_MOESM2_ESM.pdf]

Description of Additional Supplementary Files

Supplementary Data 1: Correlation between 464 SLCs gene expression (RPKM) and 225 metabolites from 898 human cancer line compiled in CCLE database.
